# Passive Acoustic Monitoring of the Temporal Variability of Odontocete Tonal Sounds from a Long-Term Marine Observatory

**DOI:** 10.1371/journal.pone.0123943

**Published:** 2015-04-29

**Authors:** Tzu-Hao Lin, Hsin-Yi Yu, Chi-Fang Chen, Lien-Siang Chou

**Affiliations:** 1 Institute of Ecology and Evolutionary Biology, National Taiwan University, No. 1, Sec. 4, Roosevelt Road, Taipei 10617, Taiwan (R.O.C.); 2 Department of Engineering Science and Ocean Engineering, National Taiwan University, No. 1, Sec. 4, Roosevelt Road, Taipei 10617, Taiwan (R.O.C.); Virginia Commonwealth Univ, UNITED STATES

## Abstract

The developments of marine observatories and automatic sound detection algorithms have facilitated the long-term monitoring of multiple species of odontocetes. Although classification remains difficult, information on tonal sound in odontocetes (i.e., toothed whales, including dolphins and porpoises) can provide insights into the species composition and group behavior of these species. However, the approach to measure whistle contour parameters for detecting the variability of odontocete vocal behavior may be biased when the signal-to-noise ratio is low. Thus, methods for analyzing the whistle usage of an entire group are necessary. In this study, a local-max detector was used to detect burst pulses and representative frequencies of whistles within 4.5–48 kHz. Whistle contours were extracted and classified using an unsupervised method. Whistle characteristics and usage pattern were quantified based on the distribution of representative frequencies and the composition of whistle repertoires. Based on the one year recordings collected from the Marine Cable Hosted Observatory off northeastern Taiwan, odontocete burst pulses and whistles were primarily detected during the nighttime, especially after sunset. Whistle usage during the nighttime was more complex, and whistles with higher frequency were mainly detected during summer and fall. According to the multivariate analysis, the diurnal variation of whistle usage was primarily related to the change of mode frequency, diversity of representative frequency, and sequence complexity. The seasonal variation of whistle usage involved the previous three parameters, in addition to the diversity of whistle clusters. Our results indicated that the species and behavioral composition of the local odontocete community may vary among seasonal and diurnal cycles. The current monitoring platform facilitates the evaluation of whistle usage based on group behavior and provides feature vectors for species and behavioral classification in future studies.

## Introduction

Odontocetes (or toothed whales) play an important role in the marine ecosystem and use a wide range of habitat for obtaining sufficient food resources. The distribution and habitat use of odontocetes fluctuate with temporal variations in habitat and prey availability [1–3]. Thus, information on the temporal variation in their habitat use can provide insights into the dynamics of habitat function and marine community structure [4].

Study of the temporal variation in odontocete habitat use remains difficult because of weather constraints and limited visibility in the ocean. Passive acoustic monitoring (PAM) has been widely used in field surveys of all cetaceans, i.e., odontocetes and mysticetes (or baleen whales) [5]. A cabled monitoring system, such as a marine observatory, is able to collect underwater recordings in real-time [6]. Long-term recording also facilitates the examination of temporal occurrence pattern of cetaceans [2,7–9]. Several automatic detectors have been developed to search for cetacean calls from a large amount of recordings [10–15]. Through the automatic processing and long-term monitoring, a marine observatory is expected to increase our knowledge on the community ecology of odontocetes.

Toothed whales produce a series of broadband clicks with extremely short interclick intervals, termed as burst pulses, during feeding [16,17]. These whales also use whistles to identify a signaler or report their own position [18,19]. The whistle contour [20,21] and repertoire composition [22] have been identified as factors crucial for recognizing different group behaviors. However, the whistle repertoire of toothed whales exhibits high inter- and intraspecific variation [23,24]. Identifying correct species and classifying different behaviors are challenging for a marine observatory, which may encounter multiple species, because the correlations between vocalizations and group behaviors have not been established for many species. Although a database for species and behavior classification is difficult to establish, investigating the temporal change of whistle characteristics and repertoire composition can provide insights into the temporal change of species composition or group behavior in the involved monitoring location.

To detect the variability of whistle usage, contour parameters are typically extracted to examine the acoustic features of whistles [23,24]. However, manual contour extraction is labor- and cost-intensive, and thus is not possible to perform during long duration recording. Several algorithms, such as the pitch-tracking algorithm, have been used to decrease the amount of laborious work involved in contour extraction [12,13,25,26] and to provide detailed information on an individual contour and the whistle repertoire. However, the performance of automatic contour extraction highly depends on the signal-to-noise ratio (SNR) of a recorded whistle. The uncertainty of extraction accuracy is elevated when the noise level is high. In addition to contour parameters, Lin et al. used a local-max detector for extracting representative frequencies from cetacean tonal sounds [15]. The distribution of representative frequencies provides additional information on the frequency characteristics and composition of whistle repertoires. This kind of information is useful in identifying usage patterns from multiple whistles instead of a single whistle.

The whistle repertoire composition is crucial for interpreting the vocal behavior of odontocetes. The classification of whistle contours obtained from long-duration recordings can be overwhelming to human operators, and thus requires assistance from automatic classification algorithms. However, the subjective categorization can highly influence the automatic classification result based on a supervised method, and some distinct whistle types might be ignored if only few classes are adopted. An alternative would be to perform an unsupervised classification on a considerable number of tonal sounds. This involves measuring the acoustic similarity between each contour pair and distinguishing different classes based on the clustering pattern of feature vectors [27,28].

In the present study, the local-max detector was used to detect burst pulses and whistles of odontocetes from recordings collected from a long-term marine observatory off northeastern Taiwan. The characteristics of whistle usage were analyzed based on the distribution of representative frequencies. The repertoire composition and sequence complexity were examined using a pitch-tracking algorithm and an unsupervised classification method. This paper discusses the potential change in the calling species and behavior of the local odontocetes among diurnal and seasonal cycles based on the temporal variation of whistle usage.

## Methods

### Study Area

The water off northeastern Taiwan is characterized by the Kuroshio Current, a warm current that flows northward along the east coast of Taiwan, and complex bathymetry, including basin and ridge. This area has the highest number of whale-watching boats in Taiwan, and several toothed whale species, such as the spinner dolphin (*Stenella longirostris*), Risso’s dolphin (*Grampus griseus*), spotted dolphin (*S*. *attenuata*), bottlenose dolphin (*Tursiops truncatus*), Fraser’s dolphin (*Lagenodelphis hosei*), and false killer whale (*Pseudorca crassidens*), are frequently encountered in this area. Although some preliminary studies have investigated distribution and abundance [1], the habitat use and community ecology of the local odontocetes remain unclear. The seasonal changes of species composition and distribution are not well studied due to the severe weather conditions during winter and fall (north-eastern monsoon period).

### Marine Cable Hosted Observatory

**Fig 1:**
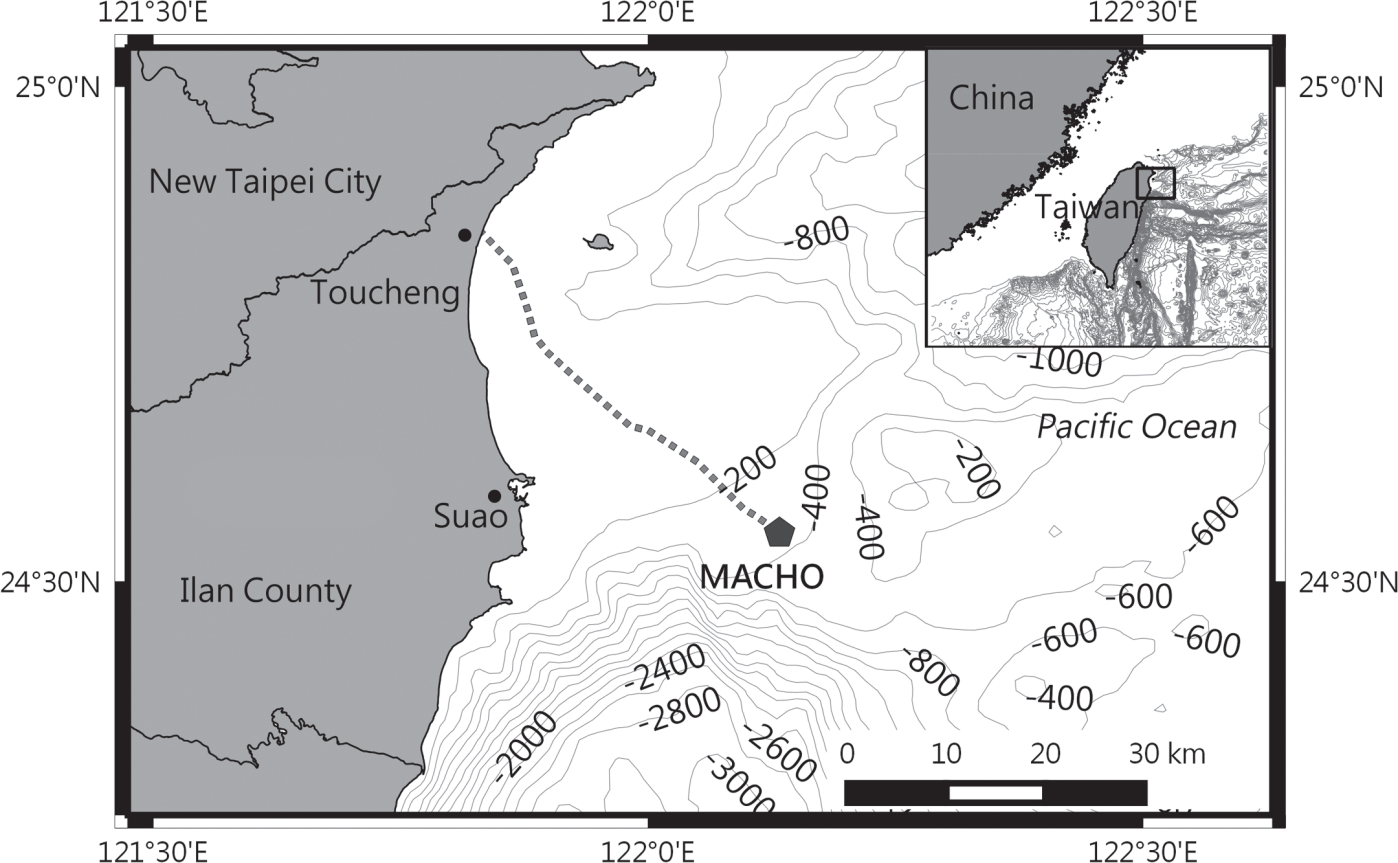
Location of the marine cable hosted observatory. The MACHO is connected with a land station at Toucheng Town through a 45-km-long submarine cable (dashed line).

In this study, the acoustic recordings were collected from the Marine Cable Hosted Observatory (MACHO). The access of MACHO recordings was permitted by the Central Weather Bureau of Taiwan (R.O.C.). Recordings are archived in the Geophysical Database Management System (http://gdms.cwb.gov.tw/index.php). The MACHO (N24°33.0’ E122°07.9’) is located on the seafloor of the Ilan Ridge, off Suao Town (Fig 1). A hydrophone (model TC-4032; Reson, Slangerup, Denmark; receiving sensitivity, –164 dB re 1 V/μPa; preamplifier gain, 10 dB; and frequency response, 10 Hz—80 kHz ± 2.5 dB) is bottom mounted at a water depth of 277 m. The recordings were collected continuously with a sampling frequency of 384 kHz and saved in the waveform audio format every 30 s. The continuous recording resulted in 64.5 GB of data per day. The acoustic monitoring system of the MACHO has a consistent systematic noise in the 3–4.5 kHz range; therefore, only the 4.5–48 kHz range was processed to prevent the influence of noise.

### Acoustic Data Processing

**Fig 2:**
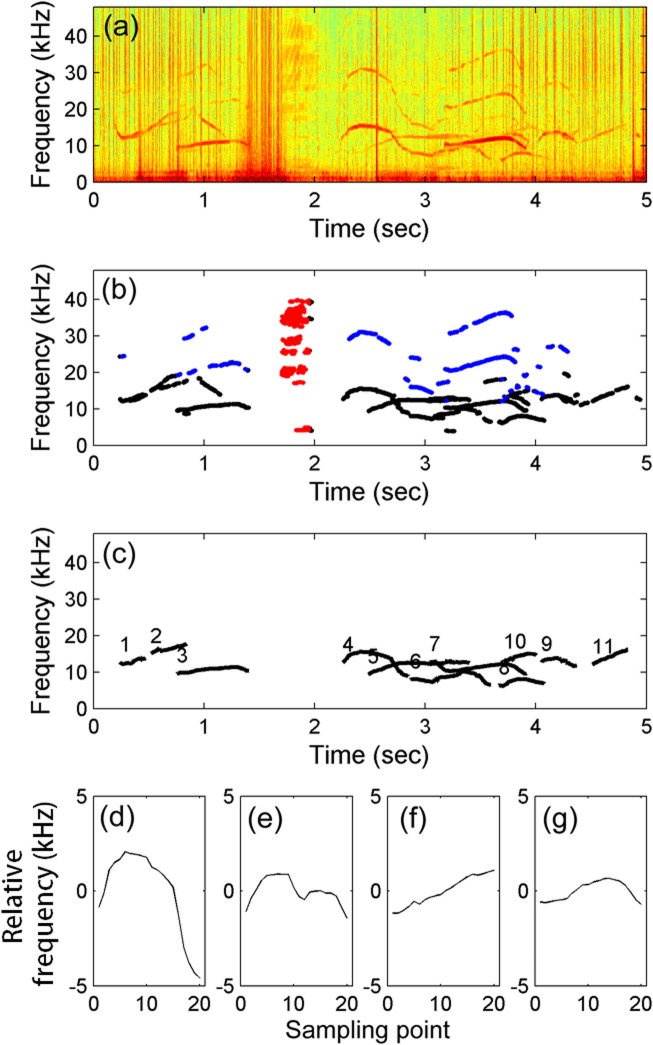
Example of automatic tonal sound detection and unsupervised classification. (a) Spectrograms produced from MACHO recording using fast Fourier transform with a Hamming window. (b) Burst pulses (red dots), harmonics (blue dots), and representative frequencies (black dots) obtained by the local-max detector. (c) Whistle contours were extracted using the pitch-tracking algorithm; different contours were labeled with different numbers. (d–g) The four whistle types were classified using the unsupervised method.

The acoustic recordings collected from October 2011 to September 2012 were examined using the automatic detection and classification algorithm, a Matlab (MathWorks, Natrick, MA)-based program, developed by Lin et al. [15,29]. This algorithm includes 4 steps: automatic detection of tonal sounds, separation of burst pulses and harmonics, contour extraction, and unsupervised classification (Fig 2).

The acoustic recordings were examined using the local-max detector. This detector can detect multiple tonal sounds simultaneously by sampling tonal spectral peaks on a pre-whitening spectrogram. The peaks were extracted when the tonality (quantified by the second derivative of the power spectral density) and the instantaneous frequency bandwidth were both higher than the threshold settings in Lin et al. [29]. Subsequently, isolated noise was removed based on the short-time transient characteristic of tonal sounds. Detection results include whistles and burst pulses with a strong tonal appearance on the spectrogram. The burst pulses were separated when the density of the tonal spectral peaks within 10 ms was higher than 0.2. The density is calculated by dividing the number of tonal spectral peaks by the area based on the number of frequency and time bins with detections. Furthermore, harmonics were separated by searching for the positive integral multiple of any detected tonal segment. The tonal segments belonging to the fundamental frequency were claimed as the representative frequencies of tonal sound and saved for further analysis. The representative frequencies were manually verified after automatic detection procedures to remove the tonal segments detected from the artificial noise, such as sonar signals and systematic noise.

Information on whistle contour is necessary for examining the whistle repertoire composition. Individual contours were extracted from the representative frequencies by using the pitch-tracking algorithm. This algorithm searched for candidate whistle fragments within a predefined frequency range and connected consecutive fragments together. When a representative frequency was located at time *t*_*i*_ and frequency *f*_*j*_, the representative frequency between *f*_*j*_ ± 1 kHz at *t*_*i+1*_ with a minimum difference in amplitude was considered the candidate contour. The terms *i* and *j* respectively represented the indices of time and frequency on the spectrogram. When no candidate contour was found at *t*_*i+a*_ (*a* represents a positive integer), the pitch-tracking algorithm extrapolated a line fitted through the previous 0.1 s of the existing track and repeated the extrapolation until *t*_*i+a*_—*t*_*i*_ was greater than 0.05 s. The candidate contour was considered to end at time *t*_*i*_. To decrease the influence of incomplete segments, contours shorter than 0.2 s were not used for further analysis.

The whistle repertoire composition was analyzed using the unsupervised classification method developed by McCowan [27]. Twenty frequency points were sampled at equal intervals from each extracted contour. Instead of calculating the correlation coefficients between all contours in a large data set, the twenty frequency points were normalized according to their mean frequency and were used as feature vectors [30]. Principle component analysis was conducted to reduce the number of collinear feature vectors. To group contours with distinguishable shapes into different clusters, only components explaining more than 90% of the variances were used as feature vectors in *k*-means cluster analysis. Clusters were grouped by minimizing the squared Euclidean distances from the observations to the centroid of each cluster. During iterative calculations of point-to-centroid distances, a new cluster comprising the point farthest from its centroid was created if all observations of a cluster were lost. The number of clusters (*k*) was selected when the mean intracluster variation (sum of point-to-centroid distances within each cluster) was reduced to 1% of the variation of the entire data set.

### Temporal Analysis of the Structural Usage of Tonal Sounds

To examine the seasonal variation in tonal sound usage, the study period was divided into 4 seasons: January–March (winter), April–June (spring), July–September (summer), and October–December (fall). The data were also separated into daytime and nighttime based on the times of sunrise and sunset (Central Weather Bureau of Taiwan) to analyze the diurnal variation. The number of seconds with detected burst pulses and whistles was measured to quantify the vocal activity of odontocetes. The factorial ANOVA function of the generalized linear model was used to test the statistical differences in the detected durations of burst pulses and whistles between two diurnal periods and among four seasons [31]. The data distribution was set as an overdispersed Poisson distribution with a log link function in the generalized linear model.

The whistle usage of odontocetes was quantified based on the mode frequency, the probability distribution of representative frequencies and whistle clusters, and the sequence complexity of whistles in each daytime and nighttime. The mode frequency represented the most commonly detected frequency, which can provide information on the frequency characteristic of calling species. Measuring mode frequency depended on the Δf of spectrograms during automatic tonal sound detection. In this study, the measuring resolution of mode frequency was 93.75 Hz.

The probability distribution of representative frequency also provides information on the frequency characteristic of calling species. The diversity index measured the evenness of representative frequency distribution. Higher diversity suggested that whistles with wider frequency ranges or different frequency characteristics were detected. The diversity index was calculated using

$$H^{\prime }=-\sum_{k=1}^{N}p_{k}\times \log p_{k},$$ 1

where *p*_*k*_ represents the proportion of the number of representative frequencies in the *k*_*th*_ frequency band (1 kHz bandwidth) in the spectrogram.

The diversity index was also employed to measure the complexity of whistle repertoire. The diversity of whistle clusters can vary according to the number of whistle clusters and the occurrence probability of each cluster. A higher number of clusters and even probability distribution can also result in higher diversity.

In addition, the complexity of whistle sequence was analyzed using information theory [32]. The zero-, first-, and second-order entropic values were calculated based on the number of whistle clusters, probability distribution of whistle clusters and the probability distribution of whistle pairs.

$$H_{0}=\log_{2}N,$$ 2

$$H_{1}=-\sum_{i=1}^{N}p_{i}\times \log_{2}p_{i},$$ 3

$$H_{2}=-\sum_{i=1}^{N}p_{i}\sum_{j=1}^{N}p_{j|i}\times \log_{2}p_{j|i},$$ 4

*N* represents the number of whistle clusters, *p*_*i*_ represents the probability of the *i*_*th*_ cluster in the whistle sequence, and *p*_*j*|*i*_ represents the conditional probability that the present whistle is the *j*_*th*_ cluster in the sequence, given that the previous whistle is the *i*_*th*_ cluster. Thereafter, the entropic slope, a measurement of whistle sequence complexity, can be determined based on the 3 entropic values and their entropic orders. An entropic slope closer to zero indicates that the structure of whistle sequence is nearly random (more complex). By contrast, a more negative entropic slope indicates that the sequence is more regular (predictable) [32,33]. Only periods with more than 3 whistle types were included in the statistical analysis regarding the whistle usage.

The principle component analysis was conducted to summarize the variation within a multivariate space by using the standardized matrix of recorded parameters (standardized by the mean and standard deviation of each parameter). The permutational multivariate analysis of variance (PERMANOVA) was conducted to test the significant difference among different periods. The homogeneity test of multivariate dispersions (PERDISP) was also used to test the significant difference in variation around the centroid among different periods. All multivariate analyses were conducted in the R Programming Environment (The R Project for Statistical Computing, http://www.r-project.org/) using the vegan package (http://vegan.r-forge.r-project.org/).

## Results

During Oct 1, 2011 to Sep 29, 2012, the MACHO had successfully transmitted recordings of 363 d and 22.73 h back to the land station. Some recording files were not received because of broken hard disk or because of other unexpected reasons. Serious data loss (> 1% recording time) occurred in 12 different days. Data recorded on these days were not included in the further analysis.

**Fig 3:**
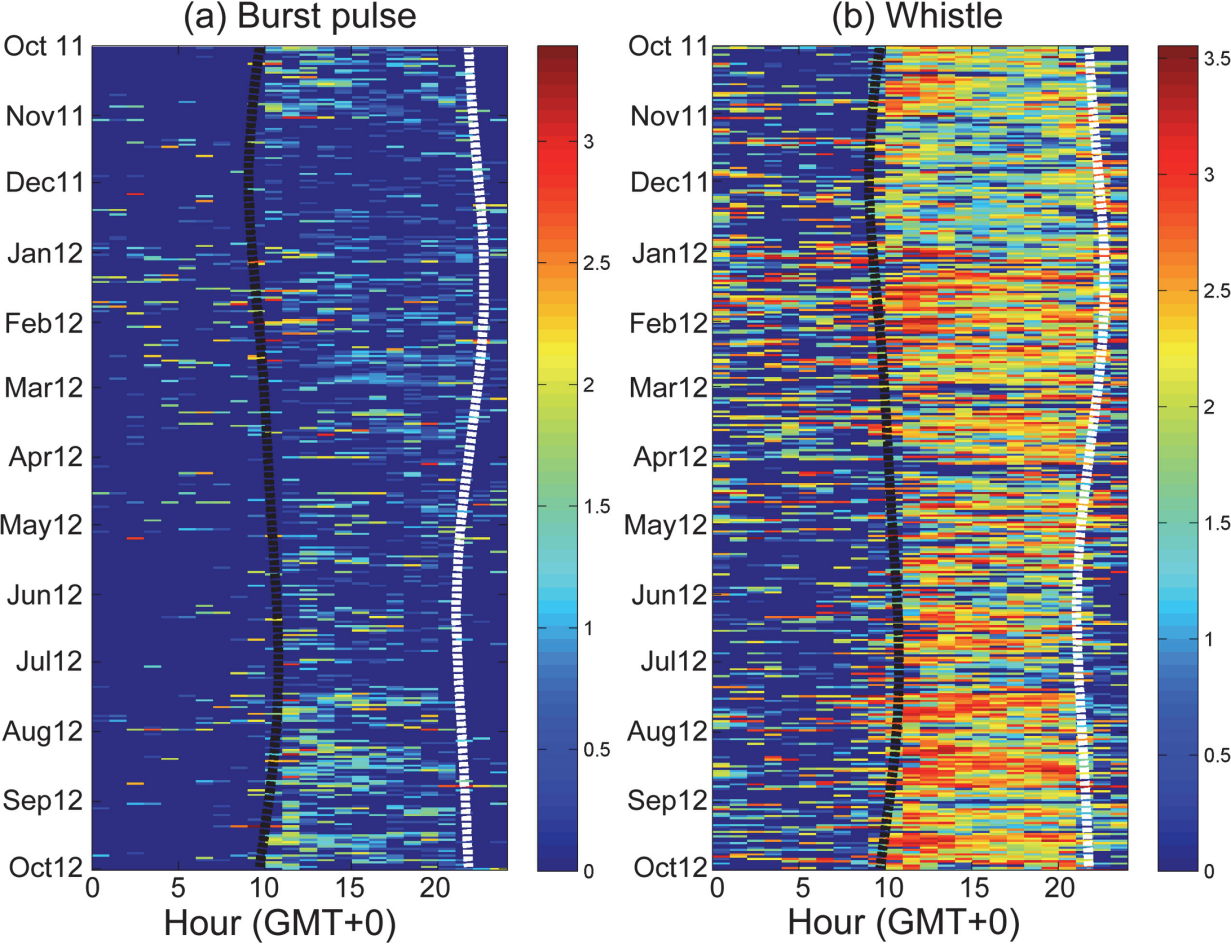
Seasonal and diurnal variation of detected durations in burst pulses (a) and whistles (b). The number of detected seconds was calculated hourly and is presented in log scale (colorbar). The black dashed line represents the time of sunset, and the white dashed line represents the time of sunrise.

The detected durations of burst pulses and whistles revealed evident seasonal as well as diurnal changes (Table 1). The detected durations of burst pulses were higher during winter and summer. By contrast, the detected durations of whistles were highest during winter (Table 2). Regarding the diurnal variation, the detected duration of both sounds revealed a similar changing pattern; the detected durations were significantly higher during the nighttime (Table 2). In addition, the detection rates of local odontocetes during 5:00 to 7:00 and 17:00 to 19:00 appeared to change with the seasonal shift of sunrise and sunset. Although no clear crepuscular behavior could be observed, the detection rates of burst pulses and whistles were slightly higher after sunset compared with the other nighttime periods (Fig 3).

**Table 1:** Results of the factorial ANOVA for seasonal and diurnal effects on the detected duration of (a) burst pulses and (b) whistles.

(a) Burst pulse			
	*df*	*Wald*	*p*
**Season**	**3**	**14.98**	**<0.01**
**Diurnal phase**	**1**	**15.31**	**<0.001**
Interaction	3	0.51	0.92
(b) Whistle			
	*df*	*Wald*	*p*
**Season**	**3**	**54.61**	**<0.001**
**Diurnal phase**	**1**	**128.37**	**<0.001**
Interaction	3	2.39	0.50

Significant effects (*p*<0.05) are bolded.

**Table 2:** The mean and standard deviation on each whistle usage parameter in the two diurnal periods and the four seasons.

			Burst pulses (sec)		Whistles (sec)		Whistle usage	Mode Frequency (kHz)		Representative frequency diversity		Whistle cluster diversity		Whistle sequence complexity	
Season	Diurnal period	N	Mean	S.D.	Mean	S.D.	N	Mean	S.D.	Mean	S.D.	Mean	S.D.	Mean	S.D.
Winter	Day	89	68	138	1944	2068	85	9.04	2.53	0.97	0.11	2.86	0.31	-0.43	0.19
	Night	89	179	337	4251	3526	89	9.92	2.73	1.05	0.08	2.96	0.24	-0.32	0.14
Spring	Day	90	33	97	988	1438	76	8.46	2.76	0.92	0.12	2.77	0.42	-0.49	0.22
	Night	90	63	113	2879	2558	88	9.01	2.95	1.00	0.11	2.80	0.34	-0.36	0.15
Summer	Day	86	78	341	1229	1707	75	11.25	2.58	1.00	0.07	2.84	0.38	-0.41	0.21
	Night	86	177	249	3471	2431	86	12.23	1.80	1.05	0.05	2.92	0.30	-0.28	0.15
Fall	Day	87	28	100	768	881	69	10.36	2.40	1.00	0.08	2.76	0.36	-0.40	0.18
	Night	87	86	267	2036	1886	87	11.67	1.94	1.07	0.04	2.82	0.30	-0.29	0.10

**Fig 4:**
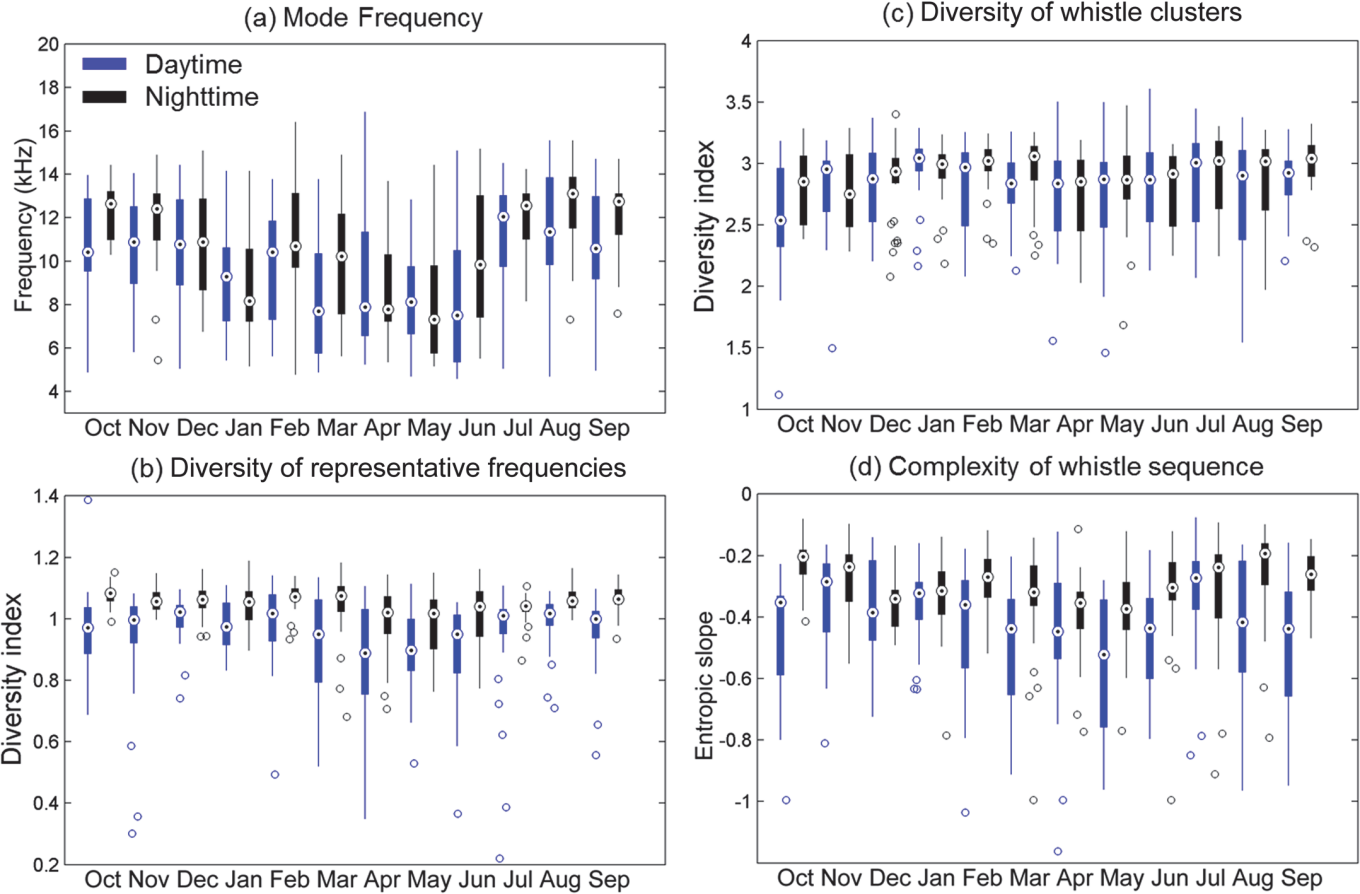
Seasonal and diurnal variation of whistle usage in mode frequency (a), diversity of representative frequencies (b), diversity of whistle clusters (c), and complexity of whistle sequence (d). The box plot shows the median (center point), interquartile range (box), minimum to maximum (error bar), and outliers (empty circles).

In addition to the detected duration, the whistle usage of local odontocetes also varied among the seasonal and diurnal cycles (Fig 4). The mode frequencies tended to be lower in winter and spring. Higher mode frequencies could also be observed during the nighttime in winter, summer, and fall, compared with those during the daytime. The diversity indices of representative frequencies in the daytime were generally lower than in the nighttime. The differences were more obvious in winter and spring. For the diversity indices of whistle clusters, the diurnal variation was not as evident. However, the seasonal variation showed that the diversity indices of whistle clusters were higher in winter. The seasonal and diurnal changing patterns of sequence complexity were much more complicated. The entropic slopes tended to be lower in the daytime, but this phenomenon can be observed only during certain months. Permutational multivariate analysis of variance revealed that the whistle usage varied significantly between day and night periods (*Pseudo-P* < 0.001) and among four seasons (*Pseudo-P* < 0.001). Furthermore, the level of data dispersion changed significantly among diurnal (*Pseudo-P* < 0.001) and seasonal cycles (*Pseudo-P* < 0.001) based on the homogeneity test of multivariate dispersions.

**Fig 5:**
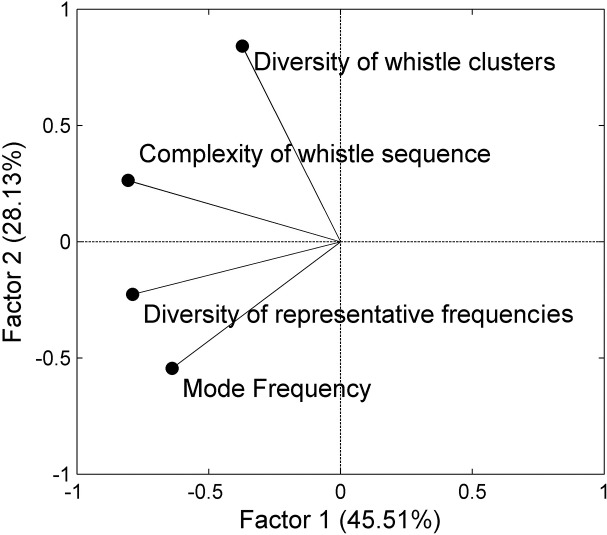
Component loadings for each whistle usage parameter. The black points represent the vectors of four whistle usage parameters on the two component factors. Factor 1 explained 45.51% of the variation of whistle usage. Factor 2 explained 28.13% of the variation of whistle usage.

**Fig 6:**
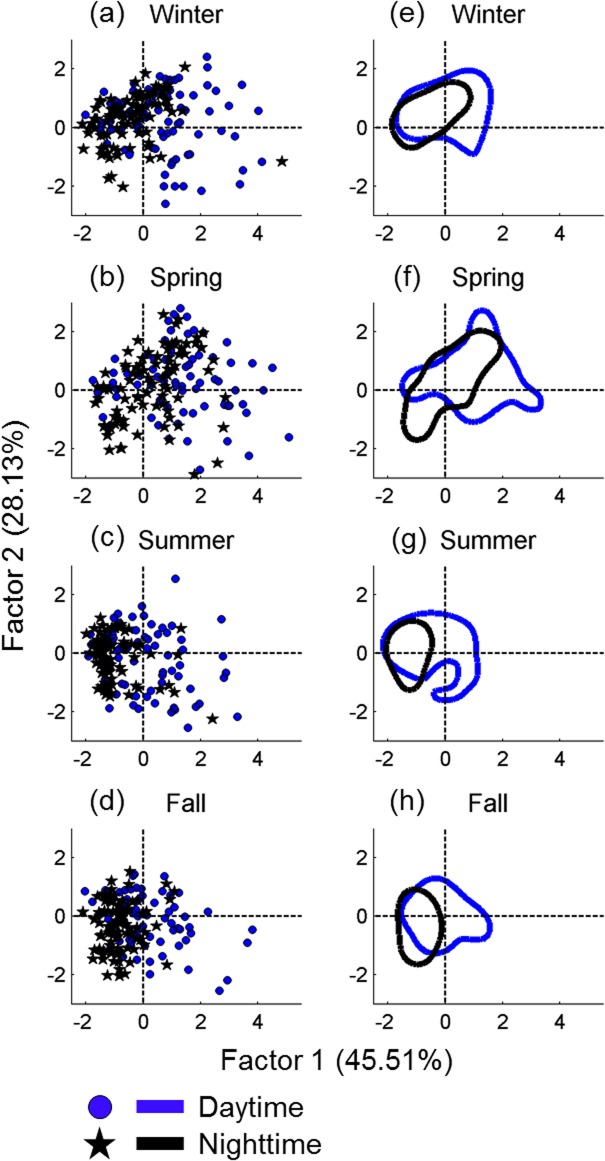
Seasonal and diurnal variation of whistle usage summarized based on the two component factors. Each data point represents one of two diurnal periods in each season after principle component analysis (a–d). The circled areas represent the 50% central areas of the two diurnal periods in each season estimated using the kernel density method (e–h).

The contribution of each parameter to the temporal variation of whistle usage was analyzed based on principle component analysis. Fig 5 shows the component loadings of the four parameters for the two principle factors. The diversity of representative frequencies and the entropic slope contributed most of the variation in Factor 1. The diversity of whistle clusters contributed most of the variation in Factor 2. The mode frequency is essential for Factors 1 and 2; however, this parameter makes a minor contribution compared to those parameters mentioned previously. According to the temporal change of whistle usage summarized based on the 2 principle factors, the diurnal change of whistle usage was clearly correlated with Factor 1. However, the seasonal change of whistle usage was correlated with both factors (Fig 6). The analysis results show that the diurnal change of whistle usage was primarily contributed by the whistle sequence complexity, diversity of representative frequencies, and mode frequency. The seasonal change of whistle usage involved all parameters. In addition, the data dispersion (the size of the 50% central area shown in Fig 6) enlarged during spring, indicating that the variations in whistle usage were greatest during spring.

## Discussion

In the present study, odontocete tonal sounds were effectively detected at the current marine observatory by using the local-max detector. In contrast to the traditional template detectors, the local-max detector can detect various types of tonal sound for facilitating the acoustic monitoring of multiple odontocete species. Applying the pitch-tracking algorithm and unsupervised classification also facilitates the examination of whistle usage. The current monitoring platform can expand knowledge on the temporal changing pattern of the local odontocete community regarding their detection rate and vocal behavior.

The performance of automatic detection is a major concern for an automatic detection algorithm. The local-max detector can detect 60% of tonal sounds recorded by the MACHO and has only a 3% false alarm rate [29], indicating that odontocete tonal sounds can be reliably examined using the current monitoring platform. To detect the variability of odontocete behavior based on whistle usage, previous studies have indicated contour parameters and the repertoire composition as predictive parameters for different surface behavioral states [20–22]. However, the SNR of a whistle recorded from a deep water marine observatory typically exhibits poor quality when the calling animal is far away from the observatory. In such a situation, contour parameters may be extracted at a lower frequency. Lin et al. [15] reported that the local-max detector could not detect some contour segments that undergo rapid changes in frequency. However, the representative frequency distribution obtained using the local-max detector was only slightly different from that obtained using the manual analysis. Therefore, instead of the maximum and minimum frequency of a contour, the mode frequency and the diversity index of representative frequencies were measured as they are less likely to be biased during a low SNR condition. Although the entire contour may not be effectively extracted, the changing pattern of composition and sequence complexity of whistle segments calculated from a large data set should reflect the relative change of the original whistle repertoire.

In contrast to contour parameters, which are typically related to the vocal behavior of a single individual, the parameters used in this study are related to the species and behavior of an entire odontocete group. Based on the few recordings collected along the east coast of Taiwan during onboard surveys, the parameters used in this study play crucial roles regarding the inter- and intraspecific variation of whistle usage. According to the loadings of the first two component factors (S1 Fig) and the factor scores of the 7 toothed whale species (S2 Fig), the interspecific variation of whistle usage can be primarily explained by mode frequencies and diversity indices of representative frequencies. Toothed whales with smaller body sizes (such as spinner and spotted dolphins) tended to produce higher mode frequencies and wider diversity indices of representative frequencies, which agree with the correlation of frequency characteristics and body size reported in previous studies [34,35]. On the contrary, the complexity of whistle repertoire and whistle sequence only explained a small percentage of the intraspecific variation. Instead, these two parameters varied between different groups of the same toothed whale species. Thus, analyzing the whistle usage in terms of the representative frequency distribution and repertoire composition may provide information related to odontocete species and group behavior.

For a long-term marine observatory, the detection rates of cetaceans can provide information on their relative change of vocal activity, and can be used to understand the temporal change of distribution and behavior [36,37]. The lower detection probability of calling animals during a period may suggest that animals either have a low calling rate or they move temporarily outside the monitoring area. However, because only a single sensor was used in this study, examining the movement of odontocetes was impossible. Although information on their movement might be lacking, detecting burst pulses and whistles can facilitate understanding the changing pattern of their vocal behavior in the study area. Burst pulses have been reported to function in social communication and feeding behavior for odontocetes [16,17,38]. Detecting burst pulses can facilitate identifying the foraging period of local odontocete communities. In addition, the long-term monitoring on whistle usage can also provide insights on temporal change of calling species and behaviors.

During the one year monitoring period, local odontocetes were primarily detected after sunset and foraged before midnight. The temporal change of whistle usage also indicated that the composition of odontocete species and group behavior possibly changed with diurnal and seasonal cycles. The higher detection rate, greater diversity of representative frequencies, and greater sequence complexity during the nighttime suggests that odontocete vocal behaviors were more complex during the nighttime. The higher mode frequencies suggest that odontocetes with smaller body sizes mainly occurred near the recording station in summer and fall. In addition, the seasonal change in the data dispersion showed that the composition of odontocete community may be more complex during spring. According to previous visual observations during spring to fall, Risso's dolphins, spinner dolphins, and spotted dolphins were sighted more often in Ilan waters during summer [1]. The preliminary results also suggested that the species diversity was higher during spring and summer, which supports the current acoustic monitoring results.

The temporal change in odontocete behavior can be correlated to prey availability [2,3]. Lee et al. [39] reported the diurnal migration of the deep scattering layer near the monitoring area. The foraging probability of odontocetes can be elevated when the deep scattering layer rises to the surface water after sunset. Furthermore, the seasonal variation in the cetacean species composition may be related to the seasonal change in the ocean environment. The northeastern Taiwan waters are highly influenced by the seasonal Kuroshio movement and upwelling [40–42]. The seasonal variation in the ocean environment may alter prey availability, and thus affect cetacean habitat use [2,3,43]. The effect of environmental change on cetacean habitat use needs to be investigated for marine conservation, and the research is only possible when data are collected for long periods and large areas.

The long-term acoustic recording system was demonstrated as a powerful tool for detecting the occurrence of odontocetes and their vocal behaviors. The current algorithm provides an objective platform for analyzing the whistle usages related to group behavior. The results can be considered as feature vectors for species and behavior classification once a database has been established. Even without a large database, the current monitoring platform still provides insights regarding the temporal change of odontocete vocal usage. Additional marine observatory networks will be constructed throughout the entire ocean. In the future, temporal variations in cetacean species composition and habitat use pattern across a large geographical area can be monitored effectively based on the network of marine observatories.

## Supporting Information

S1 Fig Component loadings for each whistle usage parameter in odontocete recordings collected from the onboard survey. During 2010–2012, 70 recordings were collected from seven odontocete species during the onboard surveys off the east coast of Taiwan. Whistle usage was examined using the same methodology as that for the MACHO recordings. The black points represent the vectors of four whistle usage parameters for the two component factors. Factor 1 explained 42.22% of the variation of whistle usage, and Factor 2 explained 37.17% of the variation of whistle usage.
(TIF)

S2 Fig Variation of whistle usage in odontocete recordings collected from the onboard survey. There were 50 recording files (total length: 3.35 hr) with an acceptable SNR of odontocete whistles. Each data point represents each recording file after principle component analysis. Whistle usage on seven species were analyzed, including bottlenose dolphins *Tursiops truncatus* (red), false killer whales *Pseudorca crassidens* (green), Fraser's dolphins *Lagenodelphis hosei* (blue), short-finned pilot whales *Globicephala macrorhynchus* (magenta), Risso’s dolphins *Grampus griseus* (cyan), spinner dolphins *Stenella longirostris* (yellow), and spotted dolphins *Stenella attenuata* (black).
(TIF)
