# Supplementary figures and images for: Passive Acoustic Monitoring of the Temporal Variability of Odontocete Tonal Sounds from a Long-Term Marine Observatory
Source: PLoS One. 2015 Apr 29;10(4):e0123943. doi: 10.1371/journal.pone.0123943 (PMC4414466; doi:10.1371/journal.pone.0123943)

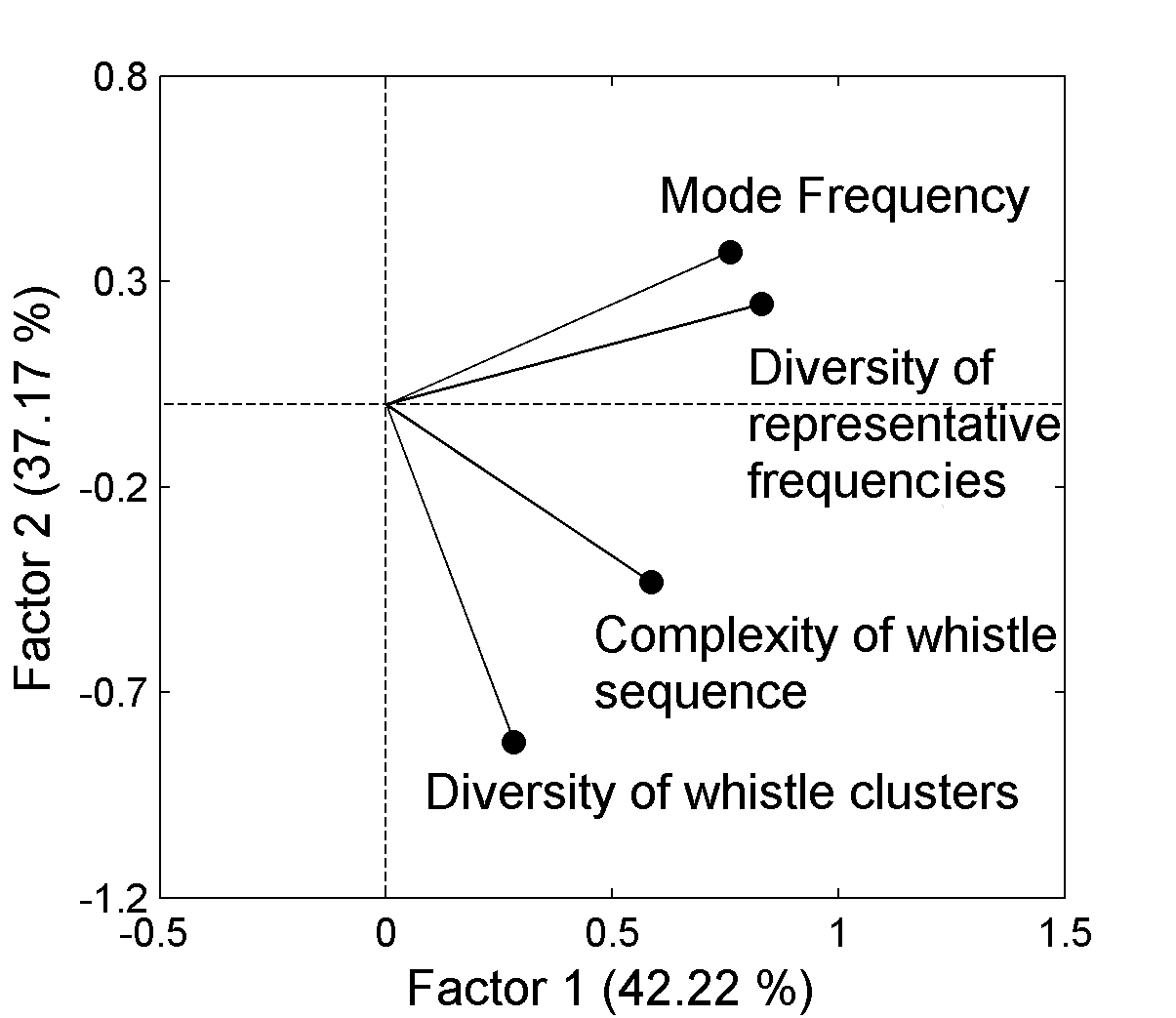

Supplement: S1 Fig — During 2010–2012, 70 recordings were collected from seven odontocete species during the onboard surveys off the east coast of Taiwan. Whistle usage was examined using the same methodology as that for the MACHO recordings. The black points represent the vectors of four whistle usage parameters for the two component factors. Factor 1 explained 42.22% of the variation of whistle usage, and Factor 2 explained 37.17% of the variation of whistle usage. (TIF) [file pone.0123943.s001.tif]

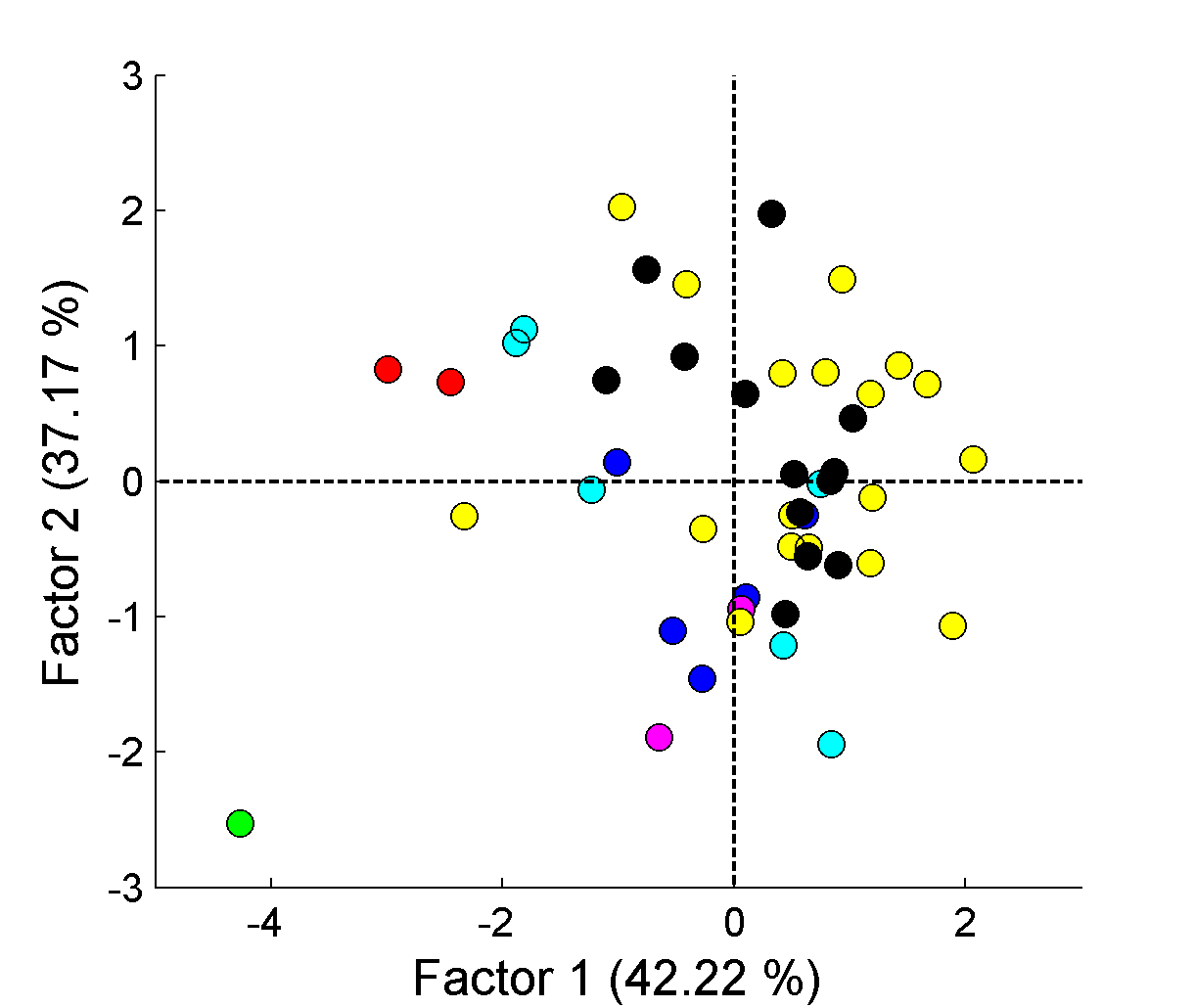

Supplement: S2 Fig — There were 50 recording files (total length: 3.35 hr) with an acceptable SNR of odontocete whistles. Each data point represents each recording file after principle component analysis. Whistle usage on seven species were analyzed, including bottlenose dolphins Tursiops truncatus (red), false killer whales Pseudorca crassidens (green), Fraser's dolphins Lagenodelphis hosei (blue), short-finned pilot whales Globicephala macrorhynchus (magenta), Risso’s dolphins Grampus griseus (cyan), spinner dolphins Stenella longirostris (yellow), and spotted dolphins Stenella attenuata (black). (TIF) [file pone.0123943.s002.tif]
